# Characteristics of pediatric patients hospitalized with COVID-19 during the third wave (omicron variant) at a referral hospital in Peru

**DOI:** 10.17843/rpmesp.2023.402.12409

**Published:** 2023-06-30

**Authors:** Giancarlo Alvarado-Gamarra, Vanessa Zarate-Campos, Jimena A. Saavedra Díaz, Renato M. Sánchez Julca, Andrea Tahua Vega, Aida Borcic, Alvaro Taype-Rondan, Luis Miguel Franchi Prato, Claudio F. Lanata, Jesús Dominguez-Rojas, Raquel Garcés-Ghilardi, Matilde Estupiñan-Vigil

**Affiliations:** 1 Clinical Pediatrics Service, Edgardo Rebagliati Martins National Hospital, Lima, Peru. Clinical Pediatrics Service Edgardo Rebagliati Martins National Hospital Lima Peru; 2 EviSalud - Evidencias en Salud, Lima, Peru. EviSalud - Evidencias en Salud Lima Peru; 3 Research Unit for the Generation and Synthesis of Health Evidence, Vice-Rectorate for Research, San Ignacio de Loyola University., Lima, Peru San Ignacio de Loyola University Research Unit for the Generation and Synthesis of Health Evidence Vice-Rectorate for Research San Ignacio de Loyola University Lima Peru; 4 Instituto de Investigación Nutricional, Lima, Peru. Instituto de Investigación Nutricional Lima Peru

**Keywords:** COVID-19, Hospitalization, Intensive Care Units, Mortality, Child, Peru

## Abstract

This study aimed to describe the characteristics of pediatric patients (28 days to 14 years of age) hospitalized with COVID-19 during the third wave of the pandemic (omicron variant) at the Hospital Nacional Edgardo Rebagliati Martins (HNERM) (Lima, Peru). In this retrospective cohort, we reviewed the medical records of 122 pediatric patients who attended HNERM between January and early April 2022 (55% male, median age: 5 years); 77.9% attended HNERM during the first month, and half of them had some comorbidity. Participants were hospitalized mainly for respiratory distress, decompensated comorbidity, and dehydration. Of the participants, 6.6% were admitted to intensive care, 4.9% to invasive mechanical ventilation, 5.7% required some vasoactive agent and 1.6% died. The most commonly used drugs were antibiotics (43.4%) and corticosteroids (27.1%). In conclusion, hospitalizations rapidly increased during the third wave, when compared to previous waves, most of them with favorable progression and with a wide empirical use of antibiotics.

## INTRODUCTION

Older adults and those with chronic comorbidities have been severely affected by COVID-19 ^1^. The involvement was mostly mild in children ^2^, with the exception of cases of pediatric multisystem inflammatory syndrome (SIM-C) associated with COVID-19 ^3^.

Several variants emerged during the pandemic. In Peru, the lambda and gamma variants (during the second wave) were associated with higher mortality and clinical involvement in adult and pediatric patients compared to the original variant ^4^. Then, the third wave of COVID-19 appeared in Peru at the beginning of 2022, in which the predominant variant was omicron (B.1.1.529) and its lineages ^4^. Several countries reported a high number of hospitalized children with omicron initially ^5^^-^^8^; however, later studies showed a lower percentage of hospitalizations and less severity compared to the delta variant ^9^^-^^11^. These reports differ, and no publications on omicron were found in Latin American pediatric centers. It is important to have data from Latin America, since what is described in other regions may not reflect what happens in countries such as Peru; due to their socioeconomic characteristics, health system, vaccination coverage, and strict public restrictions against COVID-19 ^12^.

Therefore, this study aimed to describe the characteristics of pediatric patients hospitalized with COVID-19 during the third wave (omicron variant), at the Edgardo Rebagliati Martins National Hospital (HNERM) in Lima, Peru.

**KEY MESSAGES**

Motivation for the study. There are few reports on the clinical experience of the population infected with the omicron variant of COVID-19 in Latin America, particularly in pediatric population.

Main findings. There was a rapid increase in the number of hospitalizations compared to previous waves, mainly due to respiratory conditions; most patients progressed favorably. Antibiotics and corticosteroids were the most used drugs.

Implications. Studying the characteristics of children hospitalized during the third wave of COVID-19 in Peru may increase the knowledge of how the omicron variant affects this population group, which will allow comparisons with possible new waves or diseases.

## THE STUDY

A retrospective cohort was carried out. Patients older than 28 days and younger than 14 years, hospitalized in the COVID-19 area (common ward and the HNERM intensive care unit [ICU]) were included. Patients with COVID-19 who became infected inside or outside hospital were also included.

In Peru, the first wave of COVID-19 occurred from March to December 2020, the second wave from January to June 2021, which was mostly attributed to the lambda variant ^4^, and the third wave, between the first week of January until the first days of April 2022 ^4^^,^^13^ with predominance of the omicron variant ^4^. We collected data from patients hospitalized throughout the third wave at HNERM.

The diagnosis of SARS-CoV-2 infection was made by antigen test or reverse transcriptase-polymerase chain reaction (RT-PCR). Genomic sequencing was not performed. We used the criteria from the Centers for Disease Control and Prevention (CDC) for the diagnosis of SIM-C ^14^. Data were collected from patients’ medical records (physical and electronic), from hospital admission to discharge or death.

The following variables were collected: a) epidemic wave of COVID-19, age, sex, vaccination against COVID-19, origin of the patient (emergency or intrahospital), test for diagnosis of SARS-CoV-2, comorbidities; b) reason for hospitalization, upper (croup, pharyngitis, and cold) and lower (pneumonia, wheezing, and bronchiolitis) respiratory findings based on medical diagnosis, systemic, gastrointestinal, neurological, and mucocutaneous symptoms, dysuria, cervical adenopathies, and days with symptoms before diagnosis; c) pneumonic bacterial superinfection (medical diagnosis), coronary aneurysm (by echocardiography), admission to the intensive care unit (ICU), days in ICU, requirement for invasive mechanical ventilation (IMV), days on IMV, use of high flow nasal cannula (HFNC), inotrope/vasopressor support, macrophage activation syndrome (MAS) ^15^, hospital days, death; and d) administered medications.

Data were stored in a Microsoft Excel ® file. Two researchers reviewed and debugged the final database. Statistical analysis was performed with STATA version 16 (StataCorp LP, College Station, Texas, United States). Numerical variables were reported using the mean and standard deviation or median and interquartile range (IQR) after evaluating the normal distribution of the data, which was performed by visual inspection of the histogram as well as the Shapiro-Wilk test. Categorical variables were reported by using absolute and relative frequencies.

This project was approved by the HNERM Ethics Committee (approval code: 832-2022-069). The confidentiality of the patients included in the study was preserved. The registration code for health research projects (PRISA) of this study is EI00000002918.

## FINDINGS

We found that 488 pediatric patients were hospitalized in the COVID-19 area of the HNERM from the beginning of the first wave to the end of the third wave. During the first wave (March to December 2020), 206 patients were hospitalized (42.2% of all who were hospitalized during the pandemic), 100 (20.5%) during the second wave (January to June 2021), 60 (12.3%) during the second and third wave, and 122 (25%) during the third wave (January to April 2022).

**Figure 1:**
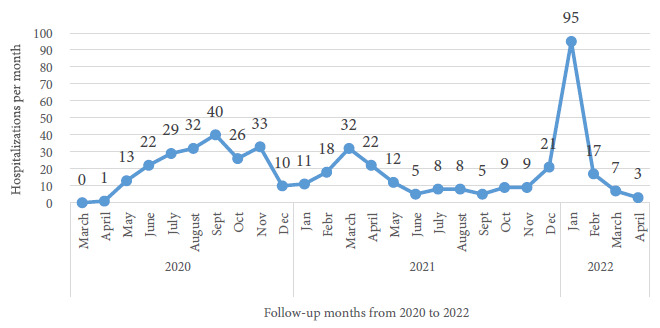
Pediatric patients hospitalized with COVID-19 per month at the Edgardo Rebagliati Martins National Hospital, 2020-2022 (n=488).

The number of hospitalizations increased rapidly during the third wave; 95 (77.9%) out of 122 patients were hospitalized only during the first month of this wave. Hospitalizations during the first months of the other waves were significantly less; one patient (0.5%) during the first month of the first wave and 11 (11.0%) during the first month of the second wave (Figure 1).

### General characteristics and comorbidities

We included 122 patients whose diagnosis was confirmed with a molecular or antigenic test, except for the 11 patients with SIM-C (these had a history of a family member with COVID-19 and met the CDC criteria). No other studies on the virus have been performed at HNERM, nor have data been collected on other possible viruses that may have coexisted. Of the total participants, 55% were male, the median age was 5 years (IQR: 2-8); most had between 6 and 11 years of age (41.8%). Only one child had received a single dose of SARS-CoV-2 vaccine, and none had previously been diagnosed with COVID-19; 8.2% became infected during hospitalization and 50.8% had some chronic comorbidity, mostly epilepsy, asthma, recurrent obstructive syndrome or chronic malnutrition (Table 1).

**Table 1:** General characteristics and comorbidities of pediatric patients hospitalized with COVID-19 during the third wave (n=122).

Characteristics	n (%)
Sex	
Male	67 (54.9)
Female	55 (45.1)
Age in years ^a^	5 (2 - 8)
Age in categories	
0 to 2 years	28 (22.9)
3 to 5 years	35 (28.7)
6 to 11 years	51 (41.8)
12 to 13 years	8 (6.6)
Origin	
Emergency	112 (91.8)
Hospitalization	10 (8.2)
COVID-19 diagnosis	
Had at least one RT-PCR positive for COVID-19	67 (54.9)
No RT-PCR positive, but yes at least one positive antigen test.	48 (39.3)
No positive RT-PCR or antigen test (symptomatic, all of them had SIM-C).	7 (5.8)
Comorbidities	
Yes	62 (50.8)
No	60 (49.2)
Comorbidity types ^b^	
Epilepsy	15 (12.3)
Asthma or recurrent obstructive syndrome	13 (10.7)
Chronic malnutrition	9 (7.4)
Leukemia	5 (4.1)
Solid tumor	5 (4.1)
Cardiopathies	5 (4.1)
Secondary neurological sequelae	3 (2.5)
Down Syndrome	4 (3.3)
Dysmorphic or genetic syndrome	3 (2.5)
Idiopathic cerebral palsy	3 (2.5)
Autism	2 (1.6)
Chronic kidney disease	2 (1.6)
Other comorbidities ^c^	28 (22.9)

RT-PCR: reverse transcriptase polymerase chain reaction, SIM-C: pediatric multisystem inflammatory syndrome associated with COVID-19.

a Median (interquartile range), ^b^ some patients may have more than one comorbidity, ^c^ cystic fibrosis, chronic lung disease, aplastic anemia, chronic liver disease, nephro-urologic malformations, chronic hematologic disorders, polytraumatized, post gastrointestinal surgery, chronic pancreatitis, congenital adrenal hyperplasia, eating disorder, and food allergy.

### Clinical profile

The main reason for hospitalization was respiratory distress (tachypnea, retractions, or oxygen saturation ≤92%) in 48.4% of patients. In addition, 14.0% were hospitalized for decompensated comorbidity, 13.1% for dehydration, 9% for SIM-C (6 had KD phenotype without shock, 3 had KD phenotype with shock, and 2 had fever and inflammation phenotype), and 15.6% for other causes (Table 2).

**Table 2:** Reason for hospitalization in the COVID-19 area and clinical manifestations of pediatric patients during the third wave (n=122).

Characteristics	n (%)
Reason for hospitalization	59 (48.4)
Respiratory distress: tachypnea, shortness of breath, or oxygen saturation ≤92%	
Decompensated comorbidity	17 (14.0)
Dehydration	16 (13.1)
SIM-C	11 (9.0)
Isolation ^a^	9 (7.4)
Febrile syndrome (without apparent origin)	6 (4.9)
Seizure	2 (1.6)
Other ^b^	2 (1.6)
Had COVID-19 symptoms prior to admission	115 (94.3)
Upper respiratory tract findings	37/115 (32.2)
Croup ^c^	7/115 (6.1)
Pharyngitis ^c^	13/115 (11.3)
Cold ^c^	17/115 (14.8)
Low respiratory tract findings ^d^	33/115 (28.7)
Pneumonia ^c^	19/115 (16.5)
Wheezing ^c^	13/115 (11.3)
Bronchiolitis ^c^	9/115 (7.8)
Other symptoms and findings ^d^	
Fever	88/115 (76.5)
Gastrointestinal symptomatology (vomiting, nausea, diarrhea, or abdominal pain)	55/115 (47.8)
Neurological symptomatology (consciousness disorder, irritability, or seizures)	27/115 (23.5)
Mucocutaneous symptomatology (rash, conjunctivitis, oral mucosal changes and lips).	7/115 (6.1)
Other (dysuria, cervical lymphadenopathy)	3/115 (2.6)
Pneumonic bacterial superinfection	8/115 (7.0)
Days with symptoms before diagnosis ^e^	(1-5)

ICU: intensive care unit, SIM-C: pediatric multisystemic inflammatory syndrome associated with COVID-19.

a Oncology patients with nosocomial infection, seven asymptomatic and two with upper respiratory symptoms, ^b^ a polytraumatized patient and another with complicated appendicitis, both diagnosed with COVID-19 on admission to the emergency room, ^c^ diagnosis based on medical criteria. Pneumonia: clinical and radiological diagnosis, ^d^ one or more findings or symptoms may coexist per patient, ^e^ median (interquartile range).

Most patients had some symptom of COVID-19, mostly fever, gastrointestinal symptoms (vomiting, nausea, diarrhea, or abdominal pain), and respiratory symptoms (upper and lower respiratory tract). Seven were asymptomatic (cancer patients with nosocomial infection). Regarding respiratory symptoms, seven (6.1%) had symptoms compatible with croup, wheezing was evident in 13 (11.3%) patients (half with previous asthma diagnosis), nine (7.8%) were diagnosed with bronchiolitis, 19 (16.5%) developed SARS-CoV-2 pneumonia (clinical and radiological diagnosis), and eight (7%) contracted pneumonic bacterial superinfection. Regarding neurological symptoms (23.5%), headache, consciousness disorder, irritability, and seizures were reported; mainly, eight of these had been previously diagnosed with epilepsy (Table 2).

### Treatment and unfavorable outcomes

All patients required some type of medication (analgesics or antihistamines), 53 (43.4%) received antibiotics (suspected bacterial superinfection), and 33 (27.1%) received corticosteroids, more frequently in patients with SIM-C (up to 63.6% for both medications). A patient with COVID-19 received corticosteroids for croup symptoms. Intravenous human immunoglobulin (IVIG) and acetylsalicylic acid were administered to most SIM-C patients, 63.6% and 72.7% respectively. Only one patient with severe COVID-19 received IVIG due to severe thrombocytopenia. Vasopressors were used in five patients with severe COVID-19 and two with SIM-C. Antifungals were used due to probable invasive fungal infections in immunosuppressed patients (one with leukemia and one with heart disease and malnutrition). No patient received tocilizumab, anticoagulant, ivermectin, hydroxychloroquine, colchicine, or vitamin C or D.

On the other hand, we found that the prevalence of pneumonic bacterial superinfection was higher in patients with SIM-C (18.2%). Only patients with SIM-C had MAS or coronary aneurysm, five (45.5%) and four (36.4%), respectively. The median hospitalization time was four days (IQR: 2-9), increasing to a median of six days in moderate/severe cases (IQR: 2-12) or SIM-C (IQR: 2-10). Likewise, eight (6.6%) patients were admitted to the ICU, six (4.9%) were in IMV, five (4.1%) required HFNC, and two (1.6%) patients died (due to COVID-19) (Table 3).

**Table 3:** Treatment and unfavorable outcomes in pediatric patients hospitalized due to COVID-19 during the third wave (n=122).

Characteristics	Total n=122	Condition		
		Moderate or severe respiratory n=59	SIM-C n=11	Other types ^a^ n=52
	n (%)	n (%)	n (%)	n (%)
Age in years ^b^	5 (2-8)	4 (2-7)	4 (0.8-8)	7 (3-9)
Comorbidity	62 (50.8)	42 (71.2)	3 (27.3)	17 (32.7)
Drugs used during hospitalization ^c^				
Antibiotics	53 (43.4)	29 (49.2)	7 (63.6)	17 (32.7)
Corticoids	33 (27.1)	25 (42.4)	7 (63.6)	1 (1.9)
Intravenous human nonspecific immunoglobulin (IVIG)	8 (6.6)	1 (1.7)	7 (63.6)	0 (0.0)
Acetylsalicylic acid	8 (6.6)	0 (0.0)	8 (72.7)	0 (0.0)
Vasopressors	7 (5.7)	5 (8.5)	2 (18.2)	0 (0.0)
Antifungals	2 (1.6)	1 (1.7)	0 (0.0)	1 (1.9)
Other events during hospitalization				
Pneumonic bacterial superinfection	8 (6.6)	6 (10.2)	2 (18.2)	0 (0.0)
Macrophage activation syndrome	5 (4.1)	0 (0)	5 (45.5)	0 (0.0)
Coronary aneurysm	4 (3.3)	0 (0)	4 (36.4)	0(0.0)
Days of hospitalization in COVID-19 area ^b^	4 (2-9)	6 (2-12)	6 (2-10)	3 (2-5)
Outcomes				
Hospitalized in common ward	114 (93.4)	53 (89.8)	9(81.8)	52 (100.0)
Hospitalized in ICU	8 (6.6)	6 (10.2)	2 (18.2)	0 (0.0)
Days in ICU ^b^	7.5 (4-16)	4.5 (4-10)	1-10 ^d^	--
Invasive mechanical ventilation	6 (4.9)	5 (8.5)	1 (9.1)	0 (0.0)
Days on invasive mechanical ventilation ^b^	4 (1-8)	1 (1-8)	7 ^e^	--
High flow cannula	5 (4.1)	5 (8.5)	0 (0.0)	0 (0.0)
Death	2 (1.6)	2 (3.4)	0 (0.0)	0 (0.0)

SIM-C: Pediatric multisystemic inflammatory syndrome associated with COVID-19. ICU: Intensive care unit.

a decompensated comorbidity, dehydration, isolation, febrile syndrome, seizure, polytraumatized, complicated appendicitis, ^b^ median (interquartile range), ^c^ no patient received tocilizumab, anticoagulant, ivermectin, hydroxychloroquine, colchicine or vitamin C or D, ^d^ one patient with SIM-C was hospitalized one day in ICU, and the other for 10 days, ^e^ one patient with SIM-C remained 7 days on invasive mechanical ventilation.

Comorbidities were more frequent in patients with moderate to severe respiratory involvement. On the other hand, the median age was similar among them (Table 3).

## DISCUSSION

This study showed a rapid increase in patients hospitalized due to COVID-19 during the third wave compared to previous waves, who mostly progressed favorably (6.6% in the ICU, 4.9% in IMV, 5.7 % used vasoactive agents, and 1.6% died). Also, the number of hospitalizations was greater during the third wave than during the second wave.

The rapid increase in hospitalizations was probably due to the high number of cases that occurred during the third wave of the pandemic in Peru, which may be due to the fact that the omicron variant is very contagious and spreads quickly ^4^. In addition, other factors could have contributed, such as comorbidities (because HNERM is a national referral hospital), the lack of vaccination in children (at that moment, only people older than 12 years old were to be vaccinated), relaxation of biosecurity measures (less social distancing, non-mandatory use of masks in public places, and fewer restrictions to access them), and the probable reservoir of uninfected pediatric patients.

Consistently, other studies have reported a large increase in pediatric hospitalizations ^5^^-^^8^, but studies that analyzed the delta variant reported fewer hospitalizations: 1.76% ^9^, 2.2% ^10^, and 10% ^16^ in the United States, and 2.3% ^11^ in Spain; as well as fewer emergency admissions, ICU admissions and IMV requirements ^9^^-^^11^.

Most hospitalized patients were male, which is similar to what was reported by other studies ^5^^,^^9^^,^^11^^,^^17^; on the other hand, most were school-age children, which differs with other studies that reported a higher frequency of hospitalizations in children under one year of age ^5^^,^^9^^,^^11^. We found that the median age was similar according to the clinical involvement (respiratory and SIM-C). Regarding comorbidities, most previous research reports patients without chronic diseases ^9^^,^^10^^,^^17^; studies similar to ours report a predominance of these as well ^5^. Those patients with moderate to severe respiratory involvement had a higher frequency of comorbidities. However, despite the fact that half of our patients had some comorbidity and none completed the vaccination schedule for COVID-19, most progressed favorably, similar to what has been described in other countries ^5^^,^^11^^,^^17^.

Respiratory conditions were the main reason for hospitalization, similar to what has been reported previously ^11^^,^^16^. Most studies report fever, gastrointestinal and respiratory symptoms ^5^^,^^8^^,^^11^, which is similar to our findings. Previous studies have also reported neurological involvement (7-20%) due to omicron ^5^^,^^11^^,^^16^^,^^17^, even in patients with no history of epilepsy. In our series, 27 (23.5%) patients presented neurological symptoms, but eight had a previous diagnosis of epilepsy.

Wheezing occurred in 13 (11.3%) patients; however, six had a diagnosis of asthma. Few studies have reported bronchospasm ^11^ due to omicron, therefore, more studies are required to assess whether this variant induces wheezing. Croup symptoms were also evidenced in seven (6.1%) patients. Croup symptoms in 12% have been reported in previous studies ^16^, as well as moderate and severe involvement ^17^^,^^18^, making it important to consider omicron as a differential diagnosis. On the other hand, the number of cases with SIM-C was lower than that found in our hospital during the first and second waves ^3^^,^^19^.

Ivermectin and hydroxychloroquine were not used in any case, which coincides with the evidence available from the study period ^20^^,^^21^ as well as with the evidence-based clinical practice guideline of the Institute for Technology Assessment in Health and Research (IETSI) of the social security of Peru (EsSalud) for the management of COVID-19 in children ^22^. However, broad empirical use of antibiotics was found (43.4%). This could be due to the suspicion of superinfection, which was low and probably the reason why only 7% received complete antibiotic treatment. The mostly used medication were corticosteroids (27.1%), although it is unknown whether these were used for any other indication unrelated to COVID-19.

On the other hand, unfavorable outcomes were not frequent, when compared to what was described during the first wave in HNERM ^19^. With omicron, hospital stay was shorter (median 6 vs. 10 days for respiratory involvement), ICU admissions were less (6.6% vs. 13%), vasopressors were less used (5.7% vs. 13%), fewer patients died (1.6% vs. 4%), and less IMV was required (4.9% vs. 14%). The favorable progression of patients infected with the omicron variant coincides with studies from the United States and Europe ^9^^-^^11^, which report less severity compared to delta; and also coincides with what was described in Peru (at the population level) with lower mortality in pediatric patients in the third wave ^4^. However, severe cases were reported, and greater severity has been reported by studies that compared other influenza and parainfluenza viruses ^17^.

The main strength of our study is that these results are representative of the largest EsSalud hospital, as well as the fact that the data was reviewed and filtered before being analyzed. However, being a retrospective study, some medical records may contain errors. Likewise, our results could differ from other hospitals in Lima. In addition, patients with nosocomial infection were included, but these represent only 8.9% of the total number of participants. Another limitation is that these cases occurred during the third wave in Peru, in which the omicron variant (B.1.1.529) predominated nationwide, therefore genomic sequencing was not performed ^4^.

In conclusion, hospitalizations due to COVID-19 increased rapidly during the third wave in the HNERM, when compared to previous waves; most patients were admitted due to respiratory conditions, and most progressed favorably. Antibiotics and corticosteroids were the most used drugs. Genomic and clinical surveillance of SARS-CoV-2 variants is necessary, as well as encouraging follow-up to assess post-COVID-19 syndrome in children.
