# Supplementary material for: Characteristics of pediatric patients hospitalized with COVID-19 during the third wave (omicron variant) at a referral hospital in Peru
Source: Rev Peru Med Exp Salud Publica. 2023 Jun 30;40(2):200–6. doi: 10.17843/rpmesp.2023.402.12409 (PMC10953665; doi:10.17843/rpmesp.2023.402.12409)
Supplement: Translation

# Translation: Características de los pacientes pediátricos hospitalizados con COVID-19 durante la tercera ola (variante ómicron) en un hospital de referencia del Perú

**Authors:** Giancarlo Alvarado-Gamarra, Vanessa Zarate-Campos, Jimena A. Saavedra Díaz, Renato M. Sánchez Julca, Andrea Tahua Vega, Aida Borcic, Alvaro Taype-Rondan, Luis Miguel Franchi Prato, Claudio F. Lanata, Jesús Dominguez-Rojas, Raquel Garcés-Ghilardi, Matilde Estupiñan-Vigil

**Affiliations:** 1 Servicio de Pediatría Clínica, Hospital Nacional Edgardo Rebagliati Martins, Lima, Perú.; 2 EviSalud - Evidencias en Salud, Lima, Perú.; 3 .Unidad de Investigación para la Generación y Síntesis de Evidencias en Salud, Vicerrectorado de Investigación, Universidad San Ignacio de Loyola, Lima, Perú.; 4 Instituto de Investigación Nutricional, Lima, Perú.

**Keywords:** COVID-19, Hospitalización, Unidades de Cuidados Intensivos, Mortalidad, Niño, Perú

## Abstract

El objetivo del presente estudio fue describir las características de los pacientes pediátricos (entre 28 días y 14 años) hospitalizados con COVID-19 durante la tercera ola (variante ómicron) en el Hospital Nacional Edgardo Rebagliati Martins (HNERM) (Lima, Perú). En esta cohorte retrospectiva, se revisaron las historias clínicas de 122 pacientes pediátricos que fueron atendidos en el HNERM entre enero e inicios de abril del 2022 (55% varones, mediana de edad de 5 años), 77,9% durante el primer mes, y la mitad presentaron alguna comorbilidad. Se hospitalizaron principalmente por dificultad respiratoria, comorbilidad descompensada y deshidratación. El 6,6% ingresaron a cuidados intensivos, 4,9% a ventilación mecánica invasiva, 5,7% requirieron algún vasoactivo y 1,6% fallecieron. Los fármacos más usados fueron antibióticos (43,4%) y corticoides (27,1%). En conclusión, se encontró un incremento rápido de hospitalizaciones en comparación con las olas anteriores, la mayoría con evolución favorable, y con un amplio uso empírico de antibióticos.

## INTRODUCCIÓN

Los adultos mayores y aquellos con comorbilidades crónicas fueron afectados severamente por la COVID-19 ^1^. En pediatría el compromiso fue mayormente leve ^2^, con excepción de los casos con síndrome inflamatorio multisistémico pediátrico asociado a COVID-19 (SIM-C) ^3^. 

Durante la pandemia surgieron varias variantes. En Perú, las variantes lambda y gamma (durante la segunda ola) se asociaron a mayor mortalidad y afectación clínica en pacientes adultos y pediátricos en comparación con la variante original ^4^. Luego, a inicios del 2022, se presentó la tercera ola de la COVID-19 en Perú, cuya variante predominante fue ómicron (B.1.1.529) y sus linajes ^4^. Varios países han reportado la experiencia con ómicron en pediatría e inicialmente encontraron mayor cantidad de hospitalizaciones ^5^^-^^8^; sin embargo, estudios posteriores muestran menor porcentaje de hospitalizaciones y menor severidad en comparación con la variante delta ^9^^-^^11^. Estos reportes son distintos, y no se encontraron publicaciones sobre ómicron en centros latinoamericanos pediátricos. Es importante contar con estos datos, pues lo descrito en otras regiones podría no reflejar lo que sucede en países latinoamericanos como el Perú; debido a sus características socioeconómicas, al sistema de salud, coberturas en vacunación, y restricciones públicas estrictas contra la COVID-19 ^12^. 

Por ello, el objetivo de este estudio fue describir las características de los pacientes pediátricos hospitalizados con COVID-19 durante la tercera ola (variante ómicron), en el Hospital Nacional Edgardo Rebagliati Martins (HNERM) en Lima, Perú.

**MENSAJE CLAVE**

Motivación para realizar el estudio. Existen pocos reportes sobre la experiencia clínica de la población infectada con la variante ómicron de la COVID-19 en Latinoamérica, en especial en población pediátrica.

Principales hallazgos. Se evidenció un incremento rápido de hospitalizaciones en comparación con las olas anteriores, principalmente por causa respiratoria, y mayormente con evolución favorable. Los fármacos evaluados más usados fueron los antibióticos y corticoides.

Implicancias. Estudiar las características de los niños hospitalizados en la tercera ola de la COVID-19 en Perú puede incrementar el conocimiento sobre la afectación de la variante ómicron a este grupo poblacional, lo que permitirá comparaciones con posibles nuevas olas o enfermedades.

## EL ESTUDIO

Se realizó una cohorte retrospectiva. Se incluyeron a pacientes mayores de 28 días y menores de 14 años, hospitalizados en el área COVID-19 (sala común y la unidad de cuidados intensivos [UCI] del HNERM). También se incluyeron a pacientes con la COVID-19 contagiados de forma extra o intrahospitalaria.

En el Perú, la primera ola de la COVID-19 ocurrió de marzo a diciembre del 2020, la segunda ola de enero a junio del 2021, atribuida mayormente a la variante lambda ^(^^4^, y la tercera ola entre la primera semana de enero hasta los primeros días de abril del 2022 ^4^^,^^13^ con predominio de la variante ómicron ^4^. Para el presente estudio, se recolectaron los datos de los pacientes hospitalizados durante toda la tercera ola en el HNERM.

El diagnóstico de infección por SARS-CoV-2 se realizó mediante prueba antigénica o reacción en cadena de la polimerasa con transcriptasa inversa (RT-PCR). No se hizo secuenciación genómica. Para el diagnóstico del SIM-C se consideró los criterios del Centers for Disease Control and Prevention (CDC) ^14^. Los datos se recolectaron de las historias clínicas (físicas y electrónicas) de los pacientes, desde el ingreso al hospital hasta el alta o fallecimiento.

Se recogieron las siguientes variables: a) ola epidémica de COVID-19, edad, sexo, vacunación contra COVID-19, origen del paciente (de la emergencia o intrahospitalario), prueba para diagnóstico de SARS-CoV-2, comorbilidades; b) motivo de hospitalización, hallazgos respiratorios altos (crup, faringitis y resfrío) y bajos (neumonía, sibilancias, y bronquiolitis) con base en el diagnóstico médico, síntomas sistémicos, gastrointestinales, neurológicos, mucocutáneos, disuria, adenopatías cervicales, y días con síntomas antes del diagnóstico; c) sobreinfección bacteriana neumónica (diagnóstico médico), aneurisma de coronarias (por ecocardiografía), ingreso a unidad de cuidados intensivos (UCI), días en UCI, requerimiento de ventilación mecánica invasiva (VMI), días en VMI, uso de cánula de alto flujo (CAF), soporte con inótropo/vasopresor, síndrome de activación macrofágica (SAM) ^15^, días de hospitalización, muerte; y d) medicamentos administrados.

Los datos se obtuvieron en el programa Microsoft Excel **®**. Dos investigadores revisaron y depuraron la base final. Se realizó el análisis estadístico con el programa STATA versión 16 (StataCorp LP, College Station, Texas, Estados Unidos). Las variables numéricas se reportaron mediante la media y desviación estándar o mediana y el rango intercuartílico (RIC), previa evaluación de la distribución normal de los datos, que se realizó usando la inspección visual del histograma y la prueba de Shapiro-Wilk. Las variables categóricas se reportaron mediante frecuencias absolutas y relativas.

El proyecto fue aprobado por el Comité de Ética del HNERM (código de aprobación: 832-2022-069). Se respetó la confidencialidad de los pacientes incluidos en el estudio. El código de registro de proyectos de investigación en salud (PRISA) fue EI00000002918.

## HALLAZGOS

Desde la primera ola hasta el fin de la tercera ola se hospitalizaron 488 pacientes pediátricos en el área COVID-19 del HNERM. En la primera ola (marzo a diciembre del 2020) se hospitalizaron 206 (42,2% del total de hospitalizados en toda la pandemia), en la segunda ola (enero a junio del 2021) 100 (20,5%), entre la segunda y tercera ola 60 pacientes (12,3%), y en la tercera ola (enero a abril del 2022) 122 (25%).

Durante la tercera ola se evidenció un incremento rápido de hospitalizaciones, de manera que el primer mes se hospitalizaron 95 (77,9%) de los 122 pacientes. La cantidad de pacientes hospitalizados durante el primer mes fue menor en las otras olas: en la primera ola un paciente (0,5%), y en la segunda 11 (11,0%) (Figura 1).

**Figura 1:** Pacientes pediatricos hospitalizados con COVID-19 por mes en el Hospital Nacional Edgardo Rebagliati Martins, 2020-2022

(n=488).

### Características generales y comorbilidades

Los 122 pacientes incluidos tuvieron el diagnóstico confirmado con prueba molecular o antigénica, excepto los 11 pacientes con SIM-C (estos tuvieron el antecedente de contacto COVID-19 intrafamiliar y todos cumplieron los criterios de la CDC). No se ha realizado de rutina otros estudios virales en el HNERM, ni se han recolectado datos sobre otros posibles virus que pudieron coexistir. Del total, 55% fueron varones, la mediana de la edad fue 5 años (RIC: 2-8), siendo el grupo de 6 a 11 años el más frecuente (41,8%). Solo un niño había recibido una sola dosis de vacuna contra el SARS-CoV-2, y ninguno tuvo COVID-19 previamente, según la anamnesis. El 8,2% se infectaron durante la hospitalización y el 50,8% presentaron alguna comorbilidad crónica, mayormente epilepsia, asma o síndrome obstructivo recurrente o desnutrición crónica (Tabla 1).

**Tabla 1:** Características generales y comorbilidades de los pacientes pediátricos hospitalizados con COVID-19 durante la tercera ola (n=122).

Características	n (%)
Sexo	
Masculino	67 (54,9)
Femenino	55 (45,1)
Edad en años ^a^	5 (2 - 8)
Edad en categorías	
0 a 2 años	28 (22.9)
3 a 5 años	35 (28,7)
6 a 11 años	51 (41,8)
12 a 13 años	8 (6,6)
Origen	
Emergencia	112 (91,8)
Hospitalización	10 (8,2)
Diagnóstico de COVID-19	
Tuvo al menos una RT-PCR positivo para COVID-19	67 (54,9)
No tuvo RT-PCR positivo, pero sí al menos una prueba de antígeno positiva	48 (39,3)
No tuvo RT-PCR ni prueba de antígeno positivos (sintomáticos, todos ellos SIM-C)	7 (5,8)
Comorbilidad	
Sí	62 (50,8)
No	60 (49,2)
Tipos de comorbilidad ^b^	
Epilepsia	15 (12,3)
Asma o síndrome obstrutivo recurrente	13 (10,7)
Desnutrición crónica	9 (7,4)
Leucemia	5 (4,1)
Tumor sólido	5 (4,1)
Cardiopatías	5 (4,1)
Secuela neurológica secundaria	3 (2,5)
Síndrome de Down	4 (3,3)
Síndrome dismórfico o genético	3 (2,5)
Parálisis cerebral idiopática	3 (2,5)
Autismo	2 (1,6)
Enfermedad renal crónica	2 (1,6)
Otras comorbilidades ^c^	28 (22,9)

RT-PCR: reacción en cadena de la polimerasa con transcriptasa inversa, SIM-C: síndrome inflamatorio multisistémico pediátrico asociado a COVID-19.

a Mediana (rango intercuartílico), ^b^ algunos pacientes podrían tener más de una comorbilidad, ^c^ Fibrosis quística, enfermedad pulmonar crónica, anemia aplásica, enfermedad hepática crónica, malformaciones nefro-urológicas, trastornos hematológicos crónicos, politraumatizado, post operado gastrointestinal, pancreatitis crónica, hiperplasia suprarrenal congénita, trastorno alimentario, y alergia alimentaria.

### Perfil clínico

El principal motivo de hospitalización fue por dificultad respiratoria (taquipnea, tirajes, o saturación de oxígeno ≤ 92%) en el 48,4% de los pacientes. Además, 14,0% se hospitalizaron por comorbilidad descompensada, 13,1% por deshidratación, 9% por SIM-C (6 con fenotipo EK sin shock, 3 con fenotipo shock con EK, y 2 con fenotipo fiebre e inflamación), y 15,6% por otras causas (Tabla 2).

**Tabla 2:** Motivo de hospitalización en el área COVID-19 y manifestaciones clínicas de los pacientes pediátricos durante la tercera ola (n=122).

Características	n (%)
Motivo de hospitalización	
Dificultad respiratoria: taquipnea, tirajes, o saturación de oxígeno ≤92%.	59 (48,4)
Comorbilidad descompensada	17 (14,0)
Deshidratación	16 (13,1)
SIM-C	11 (9,0)
Aislamiento ^a^	9 (7,4)
Síndrome febril (sin foco aparente)	6 (4,9)
Convulsión	2 (1,6)
Otros ^b^	2 (1,6)
Presentó síntomas de COVID-19 antes del ingreso	115 (94,3)
Hallazgos respiratorios altos	37/115 (32,2)
Crup ^c^	7/115 (6,1)
Faringitis ^c^	13/115 (11,3)
Resfrío ^c^	17/115 (14,8)
Hallazgos respiratorios bajos ^d^	33/115 (28,7)
Neumonía ^c^	19/115 (16,5)
Sibilancias ^c^	13/115 (11,3)
Bronquiolitis ^c^	9/115 (7,8)
Otros síntomas y hallazgos ^d^	
Fiebre	88/115 (76,5)
Sintomatología gastrointestinal (vómitos, náuseas, diarrea, o dolor abdominal)	55/115 (47,8)
Sintomatología neurológica (trastorno de conciencia, irritabilidad, o convulsiones)	27/115 (23,5)
Sintomatología mucocutánea (exantema, conjuntivitis, cambios mucosa oral y labios)	7/115 (6,1)
Otros (disuria, adenopatías cervicales)	3/115 (2,6)
Sobreinfección bacteriana neumónica	8/115 (7,0)
Días con síntomas antes del diagnóstico ^e^	(1-5)

UCI: unidad de cuidados intensivos, SIM-C: síndrome inflamatório multisistémico pediátrico asociado a COVID-19.

a Pacientes oncológicos con contagio intrahospitalario, siete asintomáticos y dos con síntomas respiratorios altos, ^b^ un paciente politraumatizado y otro con apendicitis complicada, ambos con diagnóstico de COVID-19 al ingreso a emergencia, ^c^ diagnóstico en base a criterio médico. Neumonía: diagnóstico clínico y radiológico, ^d^ puede coexistir uno o más hallazgos o síntomas por paciente, ^e^ mediana (rango intercuartílico).

La mayoría presentó algún síntoma de la COVID-19, predominando la fiebre, sintomatología gastrointestinal (vómitos, náuseas, diarrea, o dolor abdominal), y respiratoria (tracto respiratorio alto y bajo). Siete fueron asintomáticos (pacientes oncológicos con contagio intrahospitalario). Sobre los síntomas respiratorios, siete (6,1%) presentaron síntomas compatibles con crup, en 13 (11,3%) pacientes se evidenció sibilancias (la mitad con diagnóstico previo de asma), nueve (7,8%) fueron diagnosticados de bronquiolitis, 19 (16,5%) desarrollaron neumonía por SARS-CoV-2 (diagnóstico clínico y radiológico), y ocho (7%) desarrollaron sobreinfección bacteriana neumónica. Sobre la sintomatología neurológica (23,5%), se reportó cefalea, trastorno de conciencia, irritabilidad, y convulsiones, principalmente; ocho de estos con diagnóstico previo de epilepsia (Tabla 2).

### Tratamiento y desenlaces desfavorables

Todos requirieron algún tipo de medicamento (analgésicos o antihistamínicos), 53 (43,4%) pacientes recibieron antibióticos (por sospecha de sobreinfección bacteriana), y 33 (27,1%) corticoides, con mayor frecuencia en los pacientes con SIM-C (hasta 63,6% para ambos medicamentos). Un paciente con COVID-19 recibió corticoide debido a síntomas por crup. La inmunoglobulina humana endovenosa (IGIV) y el ácido acetilsalicílico se administraron en la mayoría de los pacientes con SIM-C, 63,6% y 72,7% respectivamente. Sólo un paciente con COVID-19 severo recibió IGIV debido a trombocitopenia severa. Los vasopresores se usaron en algunos pacientes con COVID-19 severo (cinco) y SIM-C (dos). Los antifúngicos se usaron en el contexto de micosis invasora probable en pacientes inmunosuprimidos (uno con leucemia y otro con cardiopatía y desnutrición). Ningún paciente recibió tocilizumab, anticoagulante, ivermectina, hidroxicloroquina, colchicina o vitamina C o D. 

Por otro lado, se observa que la sobreinfección bacteriana neumónica fue mayor en pacientes con SIM-C (18,2%). El SAM y la aparición de aneurisma de coronarias se dio solo en pacientes con SIM-C, en cinco (45,5%) y cuatro (36,4%) pacientes, respectivamente. La mediana de tiempo de hospitalización fue de cuatro días (RIC 2-9), aumentado a una mediana de seis días en casos moderados/severos (RIC 2-12) o SIM-C (RIC 2-10). Asimismo, ocho (6,6%) pacientes ingresaron a UCI, seis (4,9%) estuvieron en VMI, cinco (4,1%) requirieron CAF, y dos (1,6%) pacientes fallecieron (por COVID-19) (Tabla 3).

Los pacientes con afectación respiratoria moderada a severa presentaron mayor frecuencia de comorbilidades. Por otro lado, la mediana de la edad fue similar entre estos (Tabla 3).

**Tabla 3:** Tratamiento y desenlaces desfavorables en pacientes pediátricos hospitalizados con COVID-19 durante la tercera ola (n=122).

Características	Total n=122	Afectación		
		Respiratoria moderada o severa n=59	SIM-C n=11	Otros tipos ^a^ n=52
	n (%)	n (%)	n (%)	n (%)
Edad en años ^b^	5 (2-8)	4 (2-7)	4 (0,8-8)	7 (3-9)
Comorbilidad	62 (50,8)	42 (71,2)	3 (27,3)	17 (32,7)
Fármacos usados durante la hospitalización ^c^				
Antibióticos	53 (43,4)	29 (49,2)	7 (63,6)	17 (32,7)
Corticoides	33 (27,1)	25 (42,4)	7 (63,6)	1 (1,9)
Inmunoglobulina humana inespecífica intravenosa (IGIV)	8 (6,6)	1 (1,7)	7 (63,6)	0 (0,0)
Ácido acetilsalicílico	8 (6,6)	0 (0,0)	8 (72,7)	0 (0,0)
Vasopresores	7 (5,7)	5 (8,5)	2 (18,2)	0 (0,0)
Antifúngicos	2 (1,6)	1 (1,7)	0 (0,0)	1 (1,9)
Otros sucesos durante la hospitalización				
Sobreinfección bacteriana neumónica	8 (6,6)	6 (10,2)	2 (18,2)	0 (0,0)
Síndrome de activación macrofágica	5 (4,1)	0 (0)	5 (45,5)	0 (0.0)
Aneurisma coronario	4 (3,3)	0 (0)	4 (36,4)	0(0,0)
Días de hospitalización en área COVID-19 ^b^	4 (2-9)	6 (2-12)	6 (2-10)	3 (2-5)
Desenlaces				
Hospitalizado en sala común	114 (93,4)	53 (89,8)	9(81,8)	52 (100,0)
Hospitalizado en UCI	8 (6,6)	6 (10,2)	2 (18,2)	0 (0,0)
Días en UCI ^b^	7,5 (4-16)	4,5 (4-10)	1-10 ^d^	--
Ventilación mecánica invasiva	6 (4,9)	5 (8,5)	1 (9,1)	0 (0,0)
Días en ventilación mecánica invasiva ^b^	4 (1-8)	1 (1-8)	7 ^e^	--
Cánula de alto flujo	5 (4,1)	5 (8,5)	0 (0,0)	0 (0,0)
Muerte	2 (1,6)	2 (3,4)	0 (0,0)	0 (0,0)

SIM-C: Síndrome inflamatorio multisistémico pediátrico asociado a COVID-19. UCI: Unidad de cuidados intensivos.

a Comorbilidad descompensada, deshidratación, aislamiento, síndrome febril, convulsión, politraumatizado, apendicitis complicada, ^b^ mediana (rango intercuartílico), ^c^ ningún paciente recibió tocilizumab, anticoagulante, ivermectina, hidroxicloroquina, colchicina o vitamina C o D, ^d^ un paciente con SIM-C se hospitalizó un día en UCI, y el otro por 10 días, ^e^ un paciente con SIM-C permaneció 7 días en ventilación mecánica invasiva.

## DISCUSIÓN

En este estudio se evidenció un incremento rápido de hospitalizaciones en el área COVID-19 durante la tercera ola en comparación con las olas anteriores, pero con evolución mayormente favorable (6,6% en UCI, 4,9% en VMI, 5,7% con uso de vasoactivos, y 1,6% fallecieron). También, se encontró mayor cantidad de hospitalizaciones en comparación con la segunda ola.

Probablemente el incremento rápido de hospitalizaciones se debió al elevado número de casos que se presentaron durante la tercera ola de la pandemia en Perú, debido a la alta contagiosidad de la variante ómicron ^(^^4^. Además, otros factores pudieron haber contribuido, como las comorbilidades (al ser un hospital de referencia nacional), la falta de vacunación en niños (para esa fecha solo aprobada en Perú para mayores de 12 años), relajo de medidas de bioseguridad (menor distanciamiento social, no obligatoriedad de uso de mascarillas en lugares públicos, y menores restricciones para acceder a estos), y probable reservorio de pacientes pediátricos no infectados.

Concordantemente, otros estudios reportaron gran aumento de hospitalizaciones en pediatría ^5^^-^^8^, pero estudios que compararon con la variante delta reportaron menor cantidad de hospitalizaciones: 1,76% ^9^, 2,2% ^10^, y 10% ^16^^)^ en Estados Unidos, y 2,3% ^11^ en España; y menor cantidad de atenciones por emergencia, UCI y de requerimiento de VMI ^9^^-^^11^.

Del total de hospitalizados, la frecuencia de varones fue discretamente mayor, similar a lo reportado en otros estudios ^5^^,^^9^^,^^11^^,^^17^, con predominio en edad escolar, aunque otros trabajos reportaron mayor frecuencia de hospitalizaciones en menores de un año ^5^^,^^9^^,^^11^. La mediana de edad fue similar según afectación clínica (respiratoria y SIM-C) en nuestro hospital. Sobre las comorbilidades, la mayoría de las investigaciones reportaron pacientes sin enfermedades crónicas ^9^^,^^10^^,^^17^, otros estudios similares al nuestro reportan predominio de estas ^5^. Aquellos pacientes con afectación respiratoria moderada a severa tuvieron mayor frecuencia de comorbilidades. Sin embargo, a pesar de que la mitad de nuestros pacientes tenía alguna comorbilidad y ninguno completaba el esquema de vacunación para la COVID-19, la mayoría presentó una evolución favorable, similar a lo descrito en otros países ^5^^,^^11^^,^^17^.

El principal motivo de hospitalización fue de origen respiratorio, similar a lo hallado en otros trabajos ^11^^,^^16^. La mayoría reporta fiebre, sintomatología gastrointestinal, y respiratoria ^5^^,^^8^^,^^11^, semejante a nuestros hallazgos. También reportan afectación neurológica (7-20%) por ómicron ^5^^,^^11^^,^^16^^,^^17^, incluso en pacientes sin antecedentes de epilepsia. En nuestra serie, 27 (23,5%) pacientes presentaron sintomatología neurológica, pero ocho tuvieron diagnóstico previo de epilepsia.

Las sibilancias se presentaron en 13 (11,3%) pacientes; sin embargo, seis tenían el diagnóstico de asma. Pocos estudios han reportado broncoespasmo ^11^ por ómicron, siendo necesario más estudios que evalúen si esta variante es inductor de sibilancias. También se evidenció sintomatología de crup en siete (6,1%) pacientes. Existen estudios que reportan sintomatología de crup en un 12% ^16^, e incluso afectación moderada y severa ^17^^,^^18^, siendo importante considerar ómicron como diagnóstico diferencial. Por otro lado, el número de casos con SIM-C fue menor a lo encontrado en nuestro hospital durante la primera y segunda ola ^3^^,^^19^.

En cuanto a los medicamentos evaluados, no se observó uso de ivermectina o hidroxicloroquina, lo que coincide con la evidencia disponible en el periodo de estudio ^20^^,^^21^ y con la guía de práctica clínica basada en evidencias del Instituto de Evaluación de Tecnologías en Salud e Investigación (IETSI) del seguro social de Perú (EsSalud) para el manejo de COVID-19 en pediatría ^22^. Sin embargo, se observó un uso empírico amplio de antibióticos (43,4%). Esto pudo deberse a la sospecha de sobreinfección, que finalmente fue baja; probablemente por ello solo un 7% recibió tratamiento antibiótico completo. Asimismo, se observó un uso alto de corticoides (27,1%), aunque no se pudo distinguir si estos fueron empleados para alguna otra indicación ajena a la COVID-19.

Por otro lado, los desenlaces desfavorables no predominaron, si se compara con lo descrito durante la primera ola en nuestro hospital ^(^^19^. Con ómicron la estancia hospitalaria fue menor (mediana de 6 vs. 10 días para afectación respiratoria), ingreso a UCI menor (6,6% vs. 13%), uso menor de vasopresores (5,7% vs. 13%), menor cantidad de fallecidos (1,6% vs. 4%), y menor requerimiento de VMI (4,9% vs. 14%). Esta evolución favorable coincide con estudios de Estados Unidos y Europa ^9^^-^^11^, que reportan menor severidad en comparación con delta; y también coincide con lo descrito en Perú (a nivel poblacional) con menor mortalidad en pacientes pediátricos en la tercera ola ^(^^4^. Sin embargo, también existen casos severos, y se ha reportado mayor severidad en comparación con otros virus tipo influenza y parainfluenza ^17^.

La principal fortaleza del estudio es que estos resultados son representativos del centro hospitalario más grande de EsSalud, y los datos fueron revisados y depurados antes de ser analizados. Sin embargo, al ser un estudio retrospectivo, es posible que existan algunos registros erróneos. Asimismo, los resultados podrían ser diferentes a otros hospitales de Lima. Además, se incluyeron a pacientes con contagio intrahospitalario, pero estos representan solo el 8,9% del total. Otra limitación es que no se realizó la secuenciación genómica, ya que estos casos ocurrieron en la tercera ola peruana, dónde predominaba la variante ómicron (B.1.1.529) a nivel nacional ^4^.

En conclusión, durante la tercera ola (variante ómicron) en el HNERM, se evidenció un incremento rápido de hospitalizaciones en el área COVID-19 en comparación con las olas anteriores, principalmente por causa respiratoria, y la mayoría con evolución favorable. Los fármacos más usados fueron los antibióticos y corticoides. Es necesaria la vigilancia genómica y clínica de las variantes del SARS-CoV-2, así como incentivar el seguimiento para evaluar el síndrome pos-COVID-19 en niños.
